# Supplementary material for: Diplodia seriata Biocontrol Is Altered via Temperature and the Control of Bacteria
Source: Microorganisms. 2024 Feb 8;12(2):350. doi: 10.3390/microorganisms12020350 (PMC10892200; doi:10.3390/microorganisms12020350)
Supplement: Supplementary file 1 [file microorganisms-12-00350-s001.zip › microorganisms-2767493-supplementary.pdf]

Supplementary 1

Text S1:

Materials and Methods: Biocontrol on Grapevine Cuttings

In a study assessing the in vivo biocontrol efficacy of the grapevine endophytic bacterium *Rhodococcus* sp. PU4 and the plant growth-promoting bacteria (PGPBs) *Pseudomonas* sp. GcR15a and *Pseudomonas* sp. AMCR2b, three *D. seriata* isolates (PUCV 2120, PUCV 2142 and PUCV 2183) were utilized. The experiment involved using 18 cm long one-year-old semi-lignified cuttings from a Cabernet Sauvignon vineyard. These cuttings were disinfected, dried and stored at 5 °C for two weeks before use. Each cutting was inoculated with bacterial suspensions, a negative control with YM medium and a positive control with tebuconazole. After inoculation, the cuttings were placed in a humid chamber at different temperatures (8 and 22 °C) and were subsequently inoculated with *D. seriata*. Two months later, the length of vascular lesions was measured and the inhibition percentage for each treatment was calculated. The study employed 270 cuttings, with six cuttings for each treatment, isolate and temperature condition.

Text S2:

Results: Biocontrol on Grapevine Cuttings

The study assessed the impact of *D. seriata* isolates on grapevine cuttings pre-inoculated with plant growth-promoting bacteria (PGPB) and endophyte biocontrol bacteria (*Pseudomonas* sp. GcR15a, *Pseudomonas* sp. AMCR2b, and *Rhodococcus* sp. PU4) at 8 and 22 °C. After two months, the vascular lesion length was measured to observe damage to the grapevine cuttings. For *D. seriata* PUCV 2120, only strain AMCR2b demonstrated a significant reduction in vascular lesion length at 8 and 22 °C compared to the negative control. Regarding *D. seriata* PUCV 2142, none of the treatments showed significant differences from the negative control at 8 and 22 °C. For *D. seriata* PUCV 2183, at 22 °C, strains AMCR2b and PU4 exhibited a significant decrease in vascular lesion length compared to the negative control, while no significant differences were observed at 8 °C.

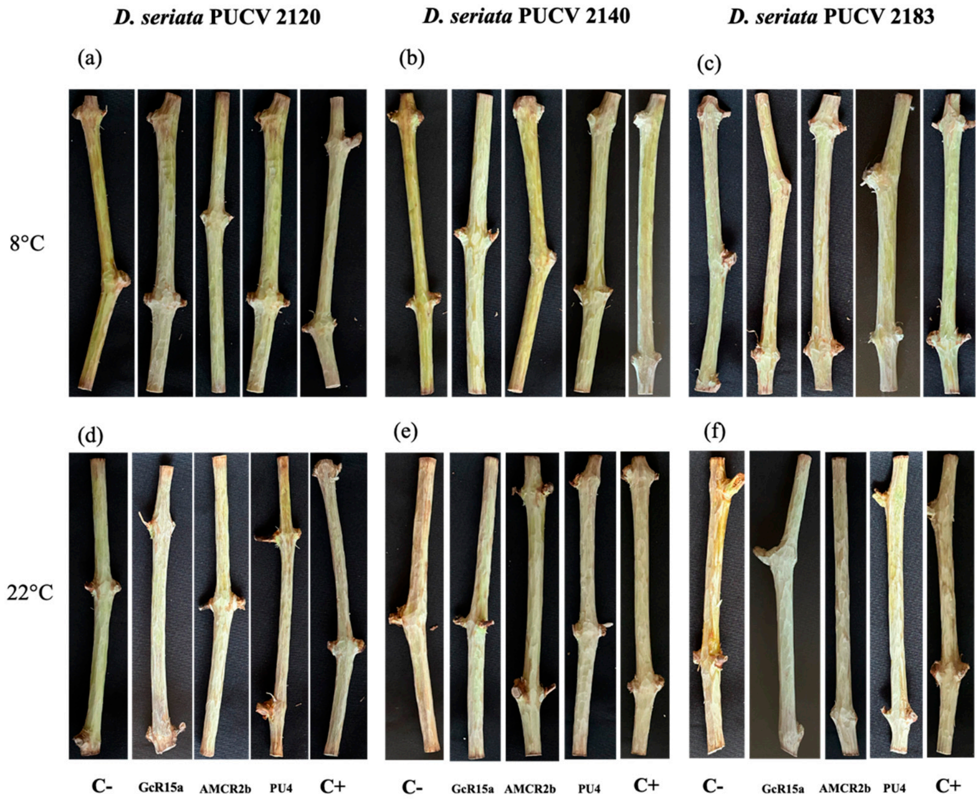

Figure S1: Effects of *Diplodia seriata* isolates grapevine pruning material pre-inoculated with PGPB and endophyte bacteria at different temperatures. (**a-c)** Effects of pre-inoculation with native bacteria on the vascular lesion length of grapevine pruning material inoculated with *D. seriata* isolates at 8 °C. (**d**–**f**) Effects of pre-inoculation with native bacteria on the vascular lesion length of grapevine pruning material inoculated with *D. seriata* isolates at 22 °C. Means with different letters indicate significant differences ($p < 0.05$). Abbreviations: C−, negative control; GcR15a, *Pseudomonas* sp. GcR15a; AMCR2b, *Pseudomonas* sp. AMCR2b; PU4, *Rhodococcus* sp. PU4; C+, positive control (tebuconazole).
